# Sagittal profile modifications in hybrid versus all screw technique in adolescent idiopathic scoliosis

**DOI:** 10.1038/s41598-020-79523-4

**Published:** 2021-01-08

**Authors:** Laura Scaramuzzo, Antonino Zagra, Giuseppe Barone, Stefano Muzzi, Leone Minoia, Marino Archetti, Fabrizio Giudici

**Affiliations:** Spine Surgery Division, 1, IRCCS Istituto Ortopedico Galeazzi, Via Riccardo Galeazzi, 4, 20161 Milan, Italy

**Keywords:** Medical research, Risk factors

## Abstract

Aim of the study was to evaluate sagittal parameters modifications, with particular interest in thoracic kyphosis, in patients affected by adolescent idiopathic scoliosis (AIS) comparing hybrid and all-screws technique. From June 2010 to September 2018, 145 patients were enrolled. Evaluation included: Lenke classification, Risser scale, coronal Cobb angle, thoracic kyphosis (TK), lumbar lordosis (LL), sagittal vertical axis (SVA), pelvic incidence (PI), pelvic tilt (PT), sacral slope (SS). Patients were divided in two groups (1 all-screws and 2 hybrid); a further division, in both groups, was done considering preoperative TK values. Descriptive and inferential statistical analysis was conducted. 99 patients were in group 1, 46 in group 2 (mean follow-up 3.7 years). Patients with a normo-kyphotic profile developed a little variation in TK (Δ pre–post = 2.4° versus − 2.0° respectively). Hyper-kyphotic subgroups had a tendency of restoring a good sagittal alignment. Hypo-kyphotic subgroups, patients treated with all-screw implants developed a higher increase in TK mean Cobb angle (Δ pre–post = 10°) than the hybrid subgroup (Δ pre–post = 5.4°) (*p* = 0.01). All-screws group showed better results in restoring sagittal alignment in all subgroups compared to hybrid groups, especially in hypo-TK subgroup, with the important advantage to give better correction on coronal plane.

## Introduction

Adolescent idiopathic scoliosis (AIS) is a progressive deformity afflicting millions of patients with a prevalence of 2–4% around the world^1^. If untreated, the progression of the deformity can lead to back pain, spinal decompensation, pulmonary function limitations and changes in appearance^1^. The threshold for surgical treatment is a major curve’s Cobb angle greater than 40°; the aim of surgical treatment is to achieve deformity correction on both coronal and sagittal plane and axial derotation while minimizing the number of fused vertebrae^2^. The restoring of sagittal balance is recognized as a critical factor in scoliosis surgery; if not properly addressed it can lead to flatback, back pain and progressive degenerative disk disease in adult age^3–5^. Therefore, assessment of preoperative sagittal flexibility and accurate intraoperative control of sagittal correction should be included in the surgical planning^6^.

Regarding surgical technique and instrumentation, various systems have been used: hooks, pedicle screws and sublaminar wires, alone or together creating hybrid systems. For some years, all-hook constructs were considered the “gold standard” treatment. Subsequently the use of pedicle screw implants for the treatment of AIS has gained much popularity, showing superior biomechanical properties^7^. Pedicle screws allow for three-dimensional deformity correction with a true derotation of the vertebrae, whereas other implants provide only posterior medialization of the spine^8,9^. At first, many surgeons thought that the potential advantage of screw fixation did not balance the risk of the technique itself (possible neurologic and vascular injury, violation of the pleura and increased radiation exposure during screw placement). However, multiple studies confirm that it is possible to perform screw fixation in the thoracic spine with both accuracy and safety^10,11^. The superiority of all-hook, all-screw or hybrid constructs is still debated^12^. Ever since, a number of Authors have shown improved curve correction using pedicle screws (alone or in hybrid implants) over all-hook constructs^13,14^.

The major limitation of all-screw implants has been at times considered to be the loss of thoracic kyphosis, a feature almost consistent in the literature with many studies asserting the hypokyphotic effect of pedicle screws in the thoracic spine^15^.

For this reason, the superior power of coronal curve correction of this technique has been thought to be at the expense of sagittal balance, leading to a higher decompensation rate. In last years, however, some studies began to deny this statement showing how pedicle screws can be used without flattening the thoracic spine^16,17^. Different studies have underlined as the restoration of a proper thoracic kyphosis depends not only on the type of anchor points but also on the applied correction maneuvers and stiffness of the rod. The use of stiffer rods, for example Crome-Cobalt ones, associated to high density construct, at least in the concave side, are able to give a good correction on coronal plane, also in more rigid curve with a satisfactory sagittal TK restoration^18,19^.

The aim of this study is to compare the modification of both sagittal and coronal balance in a cohort of 145 consecutive patients with AIS treated with either all-screw or hybrid constructs.

## Methods

The Authors retrospectively reviewed a demographic, surgical and radiographic prospectively collected database about consecutive patients who underwent surgical treatment for AIS in a single center. Inclusion criteria were: patients with AIS who underwent instrumented posterior fusion with all-screw or hybrid constructs, age between 10 to 18 years at the time of surgery, only posterior approach, absence of thoracoplasty; exclusion criteria were: main thoracolumbar/lumbar structural curve without structural thoracic curve (Lenke 5), congenital or neuromuscular scoliosis, spinal cord disorders detected on magnetic resonance imaging (MRI) scans. From June 2010 to September 2018, a total of 145 patients (31 male and 114 female) with AIS were enrolled.

Imaging evaluation consisted of pre-operative EOS X-ray, side-bending radiographs in order to determine the curve flexibility, full-spine MRI and post-operative EOS X-ray. Radiographic data were measured using a validated software (Sectra Workstation; Sectra AB) by a single expert examiner on preoperative and 4-month postoperative radiographs including: skeletal maturity (Risser grade), coronal curves Cobb angle (main curves—MC—and secondary curves—SC), thoracic kyphosis (TK), lumbar lordosis (LL), sagittal vertical axis (SVA), pelvic tilt (PT), sacral slope (SS) and the percentage of MC correction between preoperative and postoperative values (%corrMC). TK was measured from the upper endplate of T4 to the lower endplate of T12 and LL was measured from the upper endplate of L1 to the upper endplate of S1. All patients were classified according to Lenke classification^20^.

**Figure 1:**
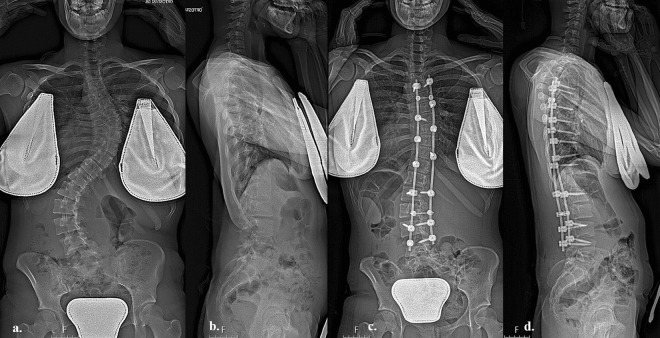
(**a**) Anteroposterior pre-operative long-cassette X-Ray of a 6 C-Adolescent Idiopathic Scoliosis in a seventeen years patients with Risser 5; (**b**) lateral pre-operative long-cassette X-ray showing hypokyphosis < 20°, (**c**) post-operative anteroposterior long-cassette X-ray showing satisfactory correction with all screw construct, (**d**) lateral post-operative long-cassette X-ray showing restoration of better kyphosis.

**Figure 2:**
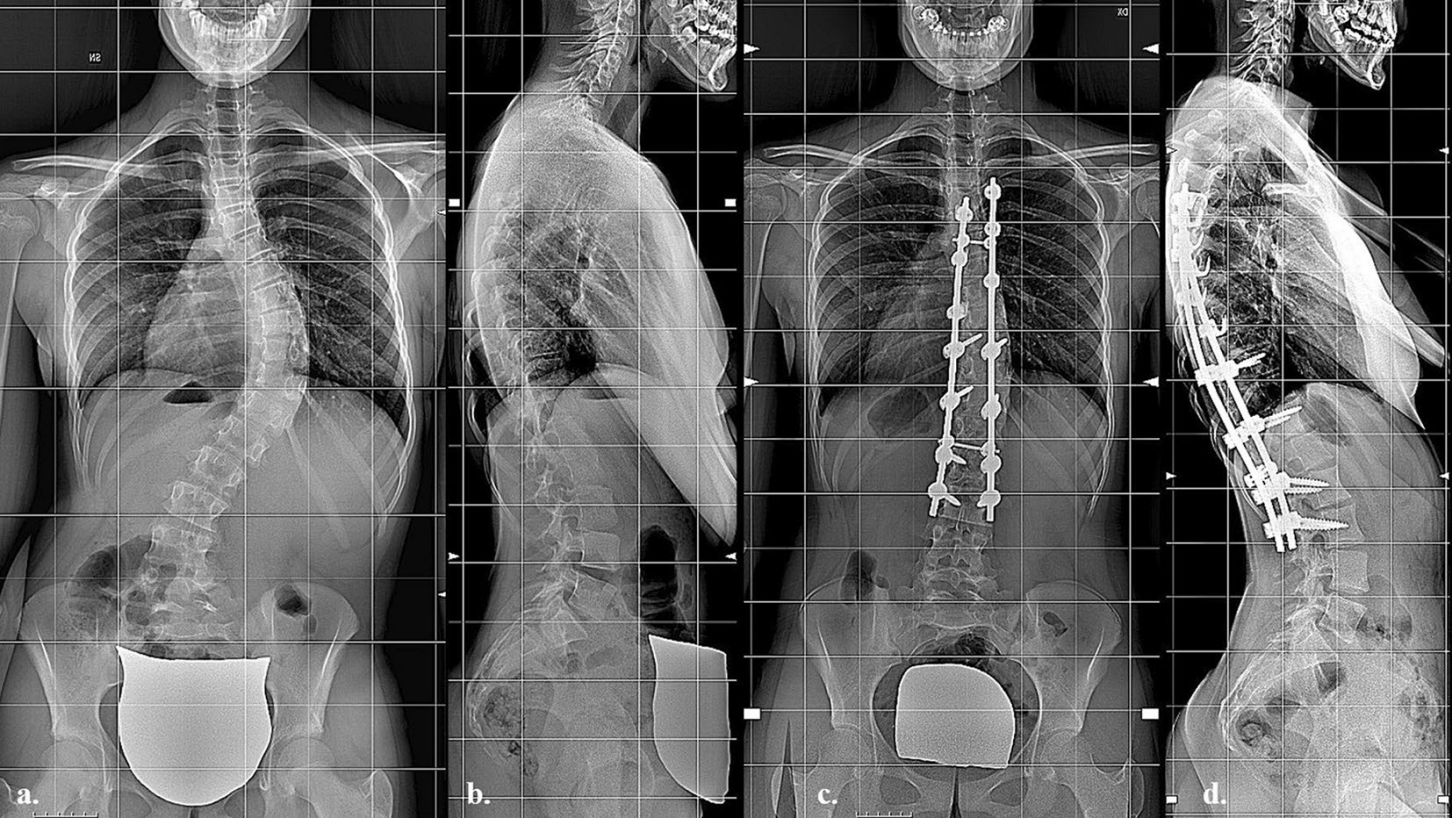
(**a**) Anteroposterior pre-operative long-cassette X-Ray of a 1C-Adolescent Idiopathic Scoliosis in a 15 years patients with Risser 3; (**b**) lateral pre-operative long-cassette X-ray showing hypokyphosis < 20°, (**c**) post-operative anteroposterior long-cassette X-ray showing satisfactory correction with hybrid construct, (**d**) lateral post-operative long-cassette X-ray showing restoration of normal kyphosis.

Patients were divided into two Groups based on the surgical technique: Group 1, 99 patients who underwent posterior instrumented fusion with all-screw technique (Fig. 1a–d); Group 2, 46 patients who underwent hybrid technique using pedicle screws and sublaminar hooks in proximal area (Fig. 2a–d). The indication to use one or the other technique depends on the preference of the surgeons involved in the study and on the increasing confidence with the use of the pedicle screws.

In order to evaluate the amount of kyphosis modification starting from preoperative baseline TK, both Groups were further divided into 3 subgroups: A (TK < 20°—hypo-kyphosis), B (20° ≤ TK ≤ 40°—normo-kyphosis), C (TK > 40°—hyper-kyphosis).

Statistical analysis was performed using IBM SPSS Statistics 21 and results were expressed using means and standard deviation (SD) or standard error (SE) for differences between means. Paired-samples *t* tests were performed to analyze pre and postoperative radiographic values while independent-samples *t* tests were conducted to compare changes in degree of curves between group 1 and group 2. All statistical tests were two-tailed and a *p* < 0.05 was considered significant.

### Surgical technique

Two senior surgeons with similar training performed all surgeries. All patients underwent posterior surgery under general anaesthesia with spinal cord monitoring of somatosensory and motor evoked potentials. Patients were placed in the prone position on a radiolucent table. After a standard midline incision, subperiostal dissection of the posterior soft tissues was performed. Before hook or screw application, inferior facetectomy was performed at each level.

### In all-screw technique

Pedicle screws were inserted with the freehand technique with the assistance of C-arm fluoroscopy. All multiaxial screws were inserted. All instrumentations were in titanium with chrome-cobalt alloy rod of 5.5 mm. All the constructs included a terminal box with a transverse connector and four screws.

### In hybrid technique

Pedicle screws were inserted in the lumbar and inferior thoracic region generally until T10. In the upper thoracic region pedicle hooks were positioned with a cephalad direction. Once the pedicle has been clearly identified, the hook is inserted with a hook holder, captive hook pusher, and mallet combined. At the superior end of the construct in the convex side, a transverse process hook with a caudal direction is positioned to obtain a stable claw construct. Also in this technique, a terminal box at the superior and inferior end of the fusion area was included.

In all patients, every level was instrumented alternatively on the concave and convex side of the curve (with a major density on the concave one). The apical vertebra was always included in the instrumented vertebrae. The laminae were thoroughly decorticated, the spinous process and the other spine constrains were removed in order to facilitate the correction manoeuvres, and the bone graft obtained from decortication was used for fusion. Correction manoeuvres implied the insertion of the rod in the concave side of the main curve as first step, previously contoured in the sagittal profile of the instrumented segment. Generally, in order to obtain a balanced spine in the sagittal profile and to prevent the remodelling of the rod during correction, a hyper kyphosis and lordosis was given to the prebent rod. A first step of correction is obtained by reducing the rod into the reduction tabs using the setscrews, in order to reach the screw head. In this way, a segmental translation of the spine to the rod was obtained. After the rod was engaged in all anchors, the rod rotation instruments were attached to the rod and the surgeon, together with the assistant, performed a global derotation of approximately 90° in the direction of the concave side. This manoeuvre allows reaching the most of correction. To obtain additional correction, especially when an axial correction is needed, a segmental derotation could be performed. At the end of the correction manoeuvres, the rods were looked inside and connected using two transverse connectors. Only in very stiff curves, additional correction with compression and distraction system was applied.

### Ethics approval and consent to participate

SpineReg Protocol (26/06/2015) and C1v1 Protocol 10/07/2015 retrospectively approved by “Comitato Etico Ospedale San Raffaele”. No experimental protocol are reported in the manuscript. The statistical analysis was conducted using IBM SPSS Statistics 21. The manuscript has been written in order to meet the International Committee of Medical Journal Editors (ICMJE) criteria for authorship. The guidelines followed for the study are in compliance with institutional and national guidelines for surgical treatment of adolescent idiopathic scoliosis.

### Informed consent

Informed consent was obtained from all subjects, and from parents/guardian/legally authorized person for patients under age of 18 years.

## Results

From the AIS database, 145 patients (31 male and 114 female) met the inclusion criteria and were enrolled. The average age was 14.4 ± 1.78 years at the time of surgery and the average Risser grade was 3.1 ± 1.6. Full demographic and intraoperative data comparing the two groups are reported in Table 1. Pre-operative and post-operative radiographic data for all patients and comparing group 1 and 2 are summarized in Table 2.

**Table 1:** Demographic and surgical data; mean [standard deviation] or (% of patients); analysis performed using IBM SPSS Statistics 21.

	All patients	Group 1	Group 2	*p*
Patients, n	145	99	46	–
Age, years	14.4 [1.78]	14.6 [1.54]	14.1 [1.31]	NS
F/M	114/31	75/24	39/7	NS
Height, cm	162.1 [7.3]	162.3 [7.4]	161.2 [7.6]	0.624
Weight, kg	50.5 [7.8]	52.7 [10.1]	53.1 [8.8]	0.539
BMI	19.7 [3.9]	20.2 [3.4]	21.2 [3.8]	0.307
Risser grade	3.1 [1.6]	3.33 [1.5]	2.45 [2.1]	0.08
Instrumented vertebrae, n	11.1 [3.7]	10.2 [2.9]	11.2 [3.2]	0.103
Selective fusion	86 (59.3)	49 (49.4)	37 (80.4)	0.029
Lenke 1 type	82 (56.5)	60 (60.6)	23 (50)	0.09
Others Lenke type	63 (43.5)	39 (39.4)	23 (50)	0.206
Implant density	1.12 [0.4]	1.10 [0.5]	1.15 [0.3]	0.607

BMI, body mass index; NS, not significant.

**Table 2:** PRE-operative radiographic parameters; mean [standard deviation]; analysis performed using IBM SPSS Statistics 21.

	All patients	Group 1	Group 2	*p*
**PRE-operative radiographic parameters; mean [standard deviation]**				
Cobb MC, °	61.5 [13.7]	63.0 [14.8]	60.0 [14.8]	NS
Cobb SC, °	42.7 [15.6]	43.9 [16.1]	40.7 [11.9]	NS
SVA, mm	− 14.5 [22.3]	− 10.2 [23.9]	− 20.7 [31.2]	0.0012
LL, °	54.1 [11.9]	54.9 [11.4]	53.9 [12.8]	NS
SS, °	38.9 [9.5]	39.2 [9.0]	38.2 [9.8]	NS
PT, °	11.0 [7.0]	10.5 [7.0]	11.8 [7.3]	NS
TK, °	23.9 [13.7]	25.7 [13.2]	22.6 [14.9]	NS
**POST-operative radiographic parameters; mean [standard deviation]**				
Cobb MC, °	26.9 [13.2]	24.6 [12.8]	30.4 [15.8]	0.01
% Correction MC	57.1 [14.3]	61.2 [14.5]	51.1 [13.7]	< 0.001
Cobb SC, °	18.9 [10.9]	16.3 [11.3]	22.7 [10.8]	0.03
SVA, mm	− 2.3 [28.9]	− 3.3 [28.3]	− 0.87 [31.1]	0.01
LL, °	49.2 [10.7]	49.6 [10.3]	48.1 [11.3]	NS
SS, °	35.7 [9.2]	35.9 [8.0]	35.7 [9.5]	NS
PT, °	12.8 [6.7]	12.5 [7.1]	13.2 [6.3]	NS
TK, °	25.4 [10.3]	28.5 [8.3]	22.0 [11.7]	0.02

MC, main curve; SC secondary curve; LL, lumbar lordosis; PT, pelvic tilt; SS, sacral slope; SVA, sagittal vertical axis; TK, thoracic kyphosis; NS, not significant.

### Comparison between pre and postoperative radiographic parameters within group 1 (all-screw)

**Figure 3:**
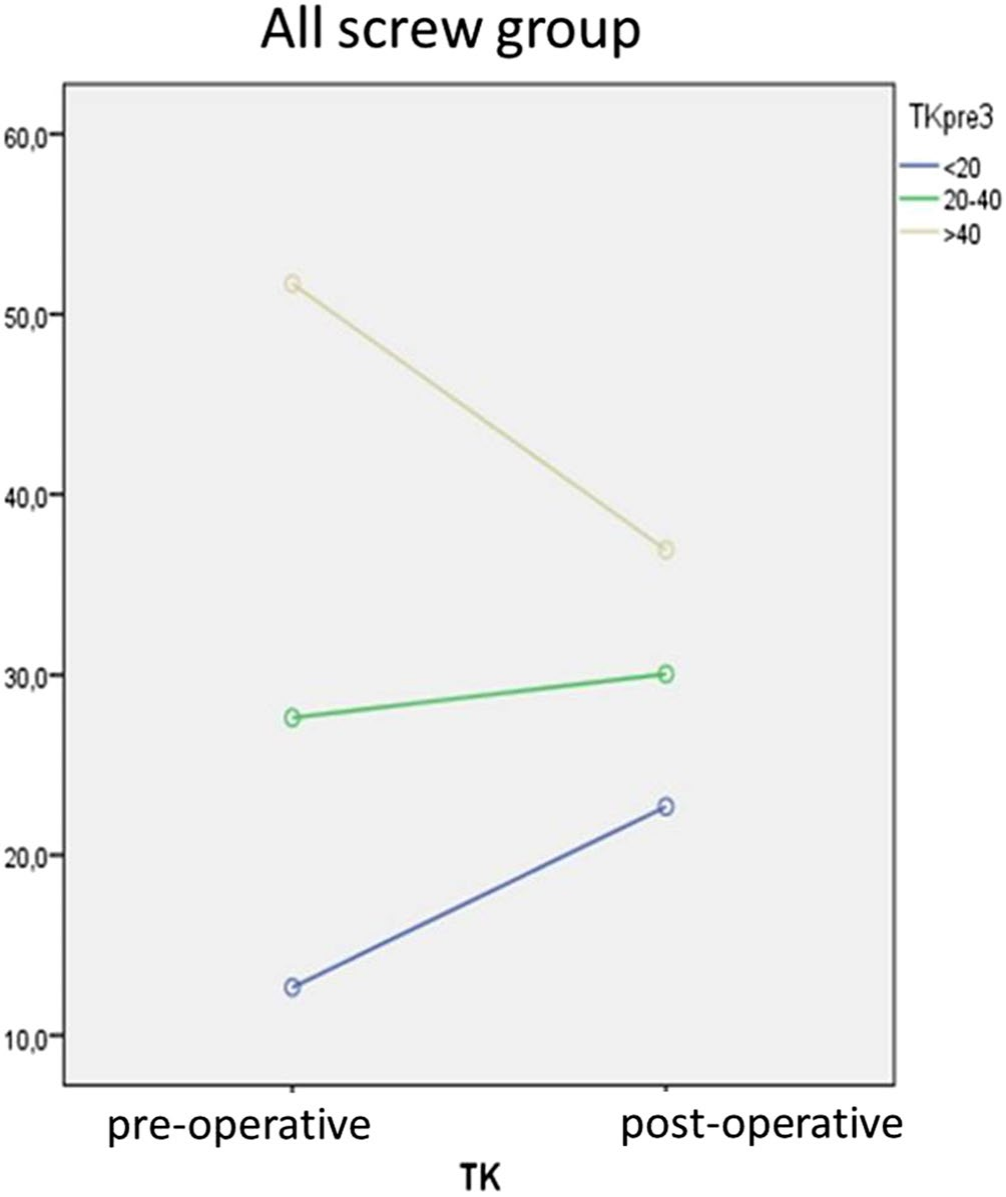
Thoracic Kyphosis correction in Group 1 (All Screw), statistical analysis performed using IBM SPSS Statistics 21.

There was a statistically significant mean difference between all preoperative and postoperative measurements. In particular, the MC decreased 38.3° ± 1.0° in average (*p* < 0.001) with a %corrMC of 61.2% ± 14.5%. Full data are reported in Table 3. The variation of TK has been studied in Table 4, considering each subgroups. There was a postoperative statistically significant mean difference within all subgroups. Figure 3 shows a tendency for all patients to reach values of normo-kyphosis (20° ≤ TK ≤ 40°).

**Table 3:** Radiographic parameters in group 2 (hybrid group); mean [standard deviation]; analysis performed using IBM SPSS Statistics 21.

	N	Preoperative	Postoperative	Δ post–pre	*p*
MC, °	46	60.0 [14.8]	30.4 [15.8]	− 29.6 [1.2]	< 0.001
SC, °	46	40.7 [11.9]	22.7 [10.8]	− 18.0 [1.3]	< 0.001
TK, °	46	22.6 [14.9]	22.0 [11.7]	− 0.5 [1.6]	0.748
LL, °	46	53.9 [12.8]	48.1 [11.3]	− 5.7 [1.6]	0.001
SVA, mm	46	− 20.7 [31.2]	− 0.87 [31.1]	19.9 [4.8]	< 0.001
PT, °	46	11.8 [7.3]	13.2 [6.3]	1.3 [0.9]	0.154
SS, °	46	38.2 [9.8]	35.7 [9.5]	− 2.5 [1.0]	0.012

LL, lumbar lordosis; MC, Cobb angle of the main curve; N, number of patients; PT, pelvic tilt; SC, Cobb angle of the secondary curve; SS, Sacral Slope; SVA, sagittal vertical axis; TK, thoracic kyphosis.

**Table 4:** Thoracic kyphosis variation in group 1 (all-screws group); patients subgroups according to preoperative thoracic kyphosis; mean [standard deviation]; analysis performed using IBM SPSS Statistics 21.

	N	Preoperative	Postoperative	Δ post–pre	*p*
**Preoperative TK**					
< 20°	32	12.7 [5.1]	22.7 [6.1]	10.0 [1.0]	< 0.001
20°–40°	55	27.6 [5.4]	30.1 [6.5]	2.4 [0.8]	0.002
> 40°	12	51.7 [8.7]	37.0 [10.4]	− 14.8 [3.4]	0.001

N, number of patients; TK, thoracic kyphosis.

### Comparison between pre and postoperative radiographic parameters within group 2 (hybrid)

**Figure 4:**
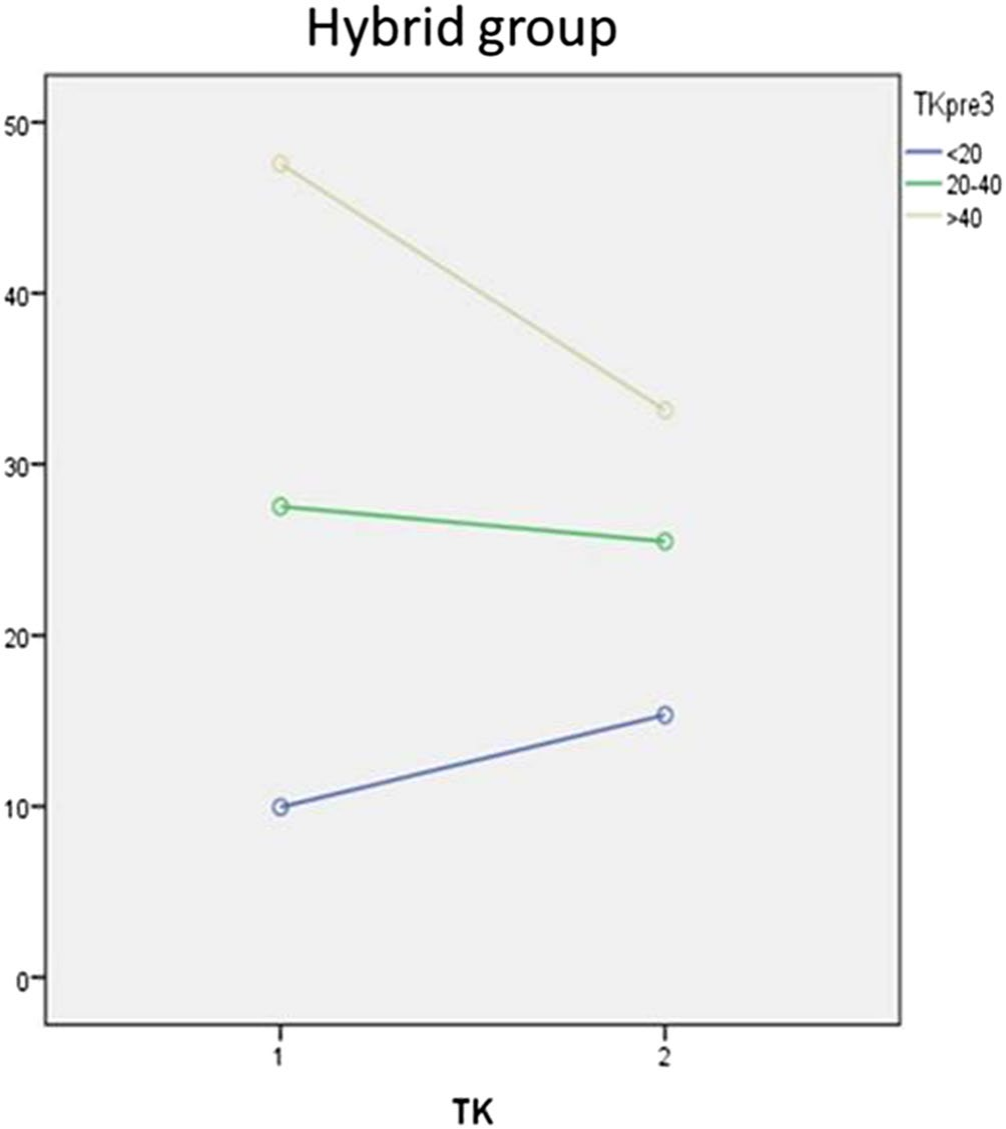
Thoracic Kyphosis correction in Group 2 (Hybrid), statistical analysis performed using IBM SPSS Statistics 21.

There was a statistically significant mean difference between all preoperative and postoperative measurements except for TK (*p* = 0.748) and PT (*p* = 0.154). The MC decreased 29.6° ± 1.2° in average (*p* < 0.001) with a %corrMC of 51.1% ± 13.7. Full data are reported in Table 5. The trend of TK has been studied in Table 6 considering each subgroups. There was a statistically significant mean difference within all subgroups with the same tendency observed in Group 1 (Figs. 3, 4).

**Table 5:** Radiographic parameters in group 1 (all-screws group); mean [standard deviation]; analysis performed using IBM SPSS Statistics 21.

	N	Preoperative	Postoperative	Δ post–pre	*p*
MC, °	99	63.0 [14.8]	24.6 [12.8]	− 38.3 [1.0]	< 0.001
SC, °	99	43.9 [16.1]	16.3 [11.3]	− 27.6 [1.1]	< 0.001
TK, °	99	25.7 [13.2]	28.5 [8.3]	2.8 [1.0]	0.006
LL, °	99	54.9 [11.4]	49.6 [10.3]	− 5.3 [1.0]	< 0.001
SVA, mm	99	− 10.2 [23.9]	− 3.3 [28.3]	6.9 [3.0]	0.021
PT, °	99	10.5 [7.0]	12.5 [7.1]	2.0 [0.6]	0.001
SS, °	99	39.2 [9.0]	35.9 [8.0]	− 3.3 [0.7]	< 0.001

LL, lumbar lordosis; MC, Cobb angle of the main curve; N, number of patients; PT, pelvic tilt; SC, Cobb angle of the secondary curve; SS, Sacral Slope; SVA, sagittal vertical axis; TK, thoracic kyphosis.

**Table 6:** Thoracic kyphosis variation in group 2 (hybrid group); patients subgroups according to preoperative thoracic kyphosis; mean [standard deviation]; analysis performed using IBM SPSS Statistics 21.

	N	Preoperative	Postoperative	Δ post–pre	*p*
**Preoperative TK**					
< 20°	21	10.0 [6.3]	15.3 [7.1]	5.4 [1.4]	0.001
20°–40°	18	27.5 [6.1]	25.5 [11.8]	− 2.0 [2.6]	0.452
> 40°	7	47.6 [7.5]	33.1 [11.3]	− 14.4 [3.5]	0.006

N, number of patients; TK, thoracic kyphosis.

### Comparison in TK trend after surgery between group 1 vs. group 2 and their respective subgroups

The differences between preoperative and postoperative TK values were compared in Table 7. There were no statistically significant mean differences among all preoperative TK values, indicating a good homogeneity between the groups at the baseline. In the comparison of postoperative TK values, the hypo-kyphotic all-screw subgroup developed a statistically significant higher increase of the kyphosis than the hybrid group (*p* < 0.001). Table 8 compared the amount of variation in TK values after surgery in hybrid and all-screw subgroups. All patients showed a trend to the normalization of sagittal alignment, whatever subgroup they belonged to. In patients treated with both all-screw and hybrid constructs, the ones with a normo-kyphotic profile developed a little variation in TK remaining in the same range of values while hyper-kyphotic subgroups had a tendency to restoring a good sagittal alignment. Among hypo-kyphotic subgroups, conversely, patients treated with all-screw implants developed a higher increase in TK mean Cobb angle than the hybrid subgroup with a statistically significant mean difference (*p* = 0.01).

**Table 7:** Difference between pre and postoperative TK values within group 1 and group 2; mean [standard deviation]; analysis performed using IBM SPSS Statistics 21.

	Δ preoperative	*p*	Δ postoperative	*p*
**Preoperative TK**				
< 20°	2.7 [1.6]	0.092	7.3 [1.8]	< 0.001
20°–40°	0.1 [1.5]	0.944	4.6 [2.9]	0.133
> 40°	4.1 [3.9]	0.311	3.8 [5.1]	0.468

TK, thoracic kyphosis.

**Table 8:** Difference between pre and postoperative amount of TK variation within Group 1 and Group 2; mean [standard deviation]; analysis performed using IBM SPSS Statistics 21.

	Δ Group 1	Δ Group 2	Difference	*p*
**Preoperative TK**				
< 20°	10.0 [6.0]	5.4 [6.4]	4.6 [1.7]	0.01
20°–40°	2.4 [5.6]	− 2.0 [11.2]	4.5 [2.8]	0.120
> 40°	− 14.8 [11.7]	− 14.4 [9.3]	− 0.3 [5.2]	0.947

TK, thoracic kyphosis.

### Comparison in TK trend in all groups and in respective subgroups considering Lenke type

The comparison in TK trend considering Lenke classification showed no statistically significant differences between all subgroups as reported in Table 9. In both groups, 1 and 2, the most patients showed a Lenke type 1 scoliosis (60/99 in group 1 and 23/46 in group 2). Despite the great difference in number all the Lenke subgroups showed a trend to normalization of TK in all subgroups considering TK pre-operative values, better represented in the all-screw group, in which is associated also to a better %MC correction.

**Table 9:** Radiographic data considering Lenke distribution mean [standard deviation]; analysis performed using IBM SPSS Statistics 21.

	Lenke 1		Lenke 2		Lenke 3		Lenke 4		Lenke 6	
	Group 1 60/99	Group 2 23/46	Group 1 9/99	Group 2 12/46	Group 1 17/99	Group 2 4/46	Group 1 4/99	Group 2 4/46	Group 1 9/99	Group 2 3/46
MC pre	56.7 [10.3]	55.3 [13.2]	66.7 [15.8]	58.8 [15.3]	77.3 [9]	70.7 [7.7]	88.5 [28.2]	88 [10]	61.6 [10.1]	52 [10]
MC post	20.1 [9]	24.5 [15.2]	31.9 [13.3]	30.1 [12.3]	34.8 [8.8]	42.7 [10.1]	52.2 [18.3]	61 [20.1]	16 [7]	19.5 [5]
p	0.01		0.04		0.01		NS		NS	
CC pre	34.8 [10.6]	34.4 [8.2]	54.6 [14.1]	39.6 [6.9]	62.5 [8.9]	65.7 [6.7]	63 [18.4]	54 [9.8]	50.1 [12]	42 [15]
CC post	10.5 [6.9]	15.8 [10]	29.7 [12.]	26 [7.1]	24.8 [8.4]	31.5 [6.7]	32.5 [12.5]	41.2 [13.4]	17.3 [10.4]	25.5 [0.5]
p	0.03		0.01		0.03		NS		0.03	
TK pre	26.4 [12.7]	18.6 [13.2]	21.3 [21.8]	24.8 [10.3]	24.8 [11.8]	40.7 [7.8]	28.7 [12.2]	25.5 [12.3]	25.6 [9.1]	22.5 [20]
TK post	29.3 [8.2]	20.7 [15.6]	27.5 [11.6]	21.1 [5.2]	26.7 [7.2]	29.2 [6.5]	28 [10.2]	26 [12.3]	27.3 [6.5]	22.5 [30]
p	NS		NS		0.01		NS		NS	

MC, main curve; CC, compensatory curve, TK, thoracic kyphosis; NS, not significant.

### Comparison in %corrMC between group 1 vs. group 2 and their respective subgroups

The comparison in %corrMC between hybrid and all-screw groups, also considering each subgroup was performed in Table 10. The patients treated with all-screw constructs, for all Lenke types, achieved a higher percentage of curve correction with a statistically significant mean difference of 10.1% (*p* < 0.001), 61.2% ± 14.5 for group 1 versus 51.1% ± 13.7 for group 2. Regarding each TK profile subgroup, all of these showed a higher %corrMC for the patients who underwent an all-screw fixation, in particular within the normo-kyphotic subgroup in which there was a statistically significant mean difference of 14% compared to the hybrid subgroup (*p* = 0.001).

**Table 10:** Percent of correction of main coronal curve between Group 1 and Group 2; all patients and patients subgroups according to preoperative thoracic kyphosis; mean [standard deviation]; analysis performed using IBM SPSS Statistics 21.

	%corrMC Group 1	%corrMC Group 2	Difference	*p*
All patients	61.2 [14.5]	51.1 [13.7]	10.1 [2.5]	< 0.001
**Preoperative TK**				
< 20°	57.9 [13.2]	53 [13.4]	4.9 [3.7]	0.196
20°–40°	62.7 [14.7]	48.7 [13.7]	14 [3.9]	0.001
> 40°	63.5 [17]	51.9 [15.7]	11.7 [7.9]	0.157

%corrMC, percent of correction between preoperative and postoperative Cobb angle of the main curve; TK, thoracic kyphosis.

## Discussion

In recent years increased attention has been set on restoring a good sagittal alignment in AIS surgery; at the same time, it is still debated which strategies and surgical techniques are the most appropriate ones to be used in order to achieve the best deformity correction. The aim of this study is to compare the impact on coronal and sagittal alignment of posterior spinal fusion in a cohort of 145 consecutive patients affected by AIS treated with all-screw or hybrid instrumentation. Considering the reported data, pedicle screw constructs seem to provide a better correction of the deformity in comparison with the hybrid technique on both coronal and sagittal plane, avoiding flatback. In medical literature several studies highlight the hypokyphotic effect of pedicle screws on thoracic spine; this issue can lead to flatback, adjacent-segment disease^21^ and loss of cervical and lumbar sagittal alignment^22^, directly affecting the clinical outcome. Lowenstein et al. found a postoperative 10° loss of thoracic kyphosis in patients treated with all-screw implants and only 3° in those who underwent hybrid technique in a cohort of 34 patients who underwent AIS surgery^23^. Kim et al. demonstrated a significant difference in 2-year postoperative kyphosis between the use of all pedicle screws compared to all hooks (17° vs. 26°, respectively)^24^. Hwang et al., analyzing a prospective database of 22 pediatric patients affected by AIS undergoing posterior spinal fusion with all-screw implants, reported a significant hypokyphotic effect on thoracic spine in 86% of patients^25^. The superiority of pedicle screws about deformity correction on coronal plane has been confirmed by many studies^14^. Recent studies hypothesizes that the hypokyphotic effect is due not exclusively to the use of pedicle screws but could be correlated to a greater extent to the adopted correction techniqu^26^. Furthermore, the three-dimensional direct segmental derotation of the vertebrae provided by pedicle screws is considered another feature that decreases thoracic kyphosis as Kota Watanabe et al. studied in a 3D simulation^27^.

In contrast with these statements, in a recent retrospectively observational study, *Srikanth Reddy *Dumpa et al. stated that screw fixation provides favorable coronal correction and improves overall sagittal parameters causing a restoration of TK in patients with hypokyphosis and hyperkyphosis preoperatively^16^.

In the present study, the Authors show that there was a normalization of TK in both hypokyphotic and hyperkyphotic subgroups while it was maintained in the normokyphotic patients. Furthermore, by the comparison of all-screw and hybrid technique, it emerges that patients with preoperative hypokyphosis who underwent pedicle screw fixation had a statistically significant higher increase than hybrid technique group (*p* < 0.01). The Authors, as a secondary goal, also evaluated the amount of coronal curve correction in both techniques. In this series, patients of Group 1 showed a statistically significant higher %corrMC in comparison with Group 2 patients (*p* < 0.001); this difference, considering each subgroups, is statistically significant among normo-kyphotic subgroups (p = 0.001). In this study the major power of coronal curve correction obtained by all-screw implants does not correlate with a hypokyphotic effect on the thoracic spine. The surgical correction technique utilized in this series could have played a fundamental role. The technique consisted of a global derotation of the spine and does not include a direct segmental derotation of the involved vertebrae, which has been recognized as a potential risk factor for developing post-operative hypokyphosis. The surgical technique may have played an important role also in providing better thoracic kyphosis in the all-screw group compared to the hybrid one. The better coronal correction with a global derotation may lead, as shown also in recent biomechanical studies^28^, to a restoration of a normal thoracic kyphosis and a normalization of the thoracolumbar junction. The better restoration of the correct antero-posterior orientation of the vertebrae included in the entire curve, obtained with all-screw constructs, especially in hypokyphotic patients, gives better result in TK restoring. The better correction force of the screws compared to hooks plays a major role to obtain the desired alignment in the sagittal and coronal plane. Another limitation ascribed to the utilization of pedicle screws in the thoracic spine for AIS correction is the rate of neurologic or vascular complications caused by misplaced screws because of the vertebral dystrophy observed in the concavity of scoliosis. In this series no patients experienced neurologic or vascular complications, even thanks to the new neuromonitoring technology utilized during surgery recently, showing that pedicle screw technique can be performed in safety, even in the thoracic spine. In addition, the use of low-density implants reduces the risk of complications and provides a more harmonic deformity correction that allows the spine to balance during years.

The main finding of this study is that posterior spinal fusion with all-screw implants in AIS surgery provides a better correction of the deformity in comparison with the hybrid technique on both coronal and sagittal plane, avoiding flatback.

There are some limitations that has to be acknowledge to this study: the nature of the study is retrospective, the follow-up period is limited, and the radiographic values are subjected to inconsistencies in positioning and measurement reliability.

The strengths of this study are one of the largest cohort of consecutive patients in single center underwent AIS surgery present in literature, the uniform of the surgeons who performed all the operations and the uniform within each of the two techniques compared in terms of screws density and derotational technique.

## Data Availability

The authors declare the availability of data and materials for further analyses.
